# The Restrained Expression of NF-kB in Renal Tissue Ameliorates Folic Acid Induced Acute Kidney Injury in Mice

**DOI:** 10.1371/journal.pone.0115947

**Published:** 2015-01-05

**Authors:** Dev Kumar, Surinder K. Singla, Veena Puri, Sanjeev Puri

**Affiliations:** 1 Department of Biochemistry, Panjab University, Chandigarh, India; 2 Centre for Systems Biology & Bioinformatics, Panjab University, Chandigarh, India; 3 Biotechnology Branch, University Institute of Engineering & Technology, Panjab University, Chandigarh, India; 4 Centre for Stem Cell & Tissue Engineering, Panjab University, Chandigarh, India; National Cancer Institute, United States of America

## Abstract

The Nuclear factor kappa-light-chain-enhancer of activated B cells (NF-kB) represent family of structurally-related eukaryotic transcription factors which regulate diverse array of cellular processes including immunological responses, inflammation, apoptosis, growth & development. Increased expression of NF-kB has often been seen in many diverse diseases, suggesting the importance of genomic deregulation to disease pathophysiology. In the present study we focused on acute kidney injury (AKI), which remains one of the major risk factor showing a high rate of mortality and morbidity. The pathology associated with it, however, remains incompletely known though inflammation has been reported to be one of the major risk factor in the disease pathophysiology. The role of NF-kB thus seemed pertinent. In the present study we show that high dose of folic acid (FA) induced acute kidney injury (AKI) characterized by elevation in levels of blood urea nitrogen & serum creatinine together with extensive tubular necrosis, loss of brush border and marked reduction in mitochondria. One of the salient observations of this study was a coupled increase in the expression of renal, relA, NF-kB2, and p53 genes and proteins during folic acid induced AKI (FA AKI). Treatment of mice with NF-kB inhibitor, pyrrolidine dithio-carbamate ammonium (PDTC) lowered the expression of these transcription factors and ameliorated the aberrant renal function by decreasing serum creatinine levels. In conclusion, our results suggested that NF-kB plays a pivotal role in maintaining renal function that also involved regulating p53 levels during FA AKI.

## Introduction

Acute Kidney Injury (AKI) is a serious and frequent clinical complication leading to a sudden loss of renal function. It is associated with a very high mortality rate affecting approximately 2 million people every year and those who survive face a higher risk for development of chronic kidney disease (CKD) [1]–[3]. Till date the, dialysis form the part of approved treatment for AKI [4]. In spite of intermittent hemodialysis (IHD) and continuous renal replacement therapy (CRRT) which are widely being used modes of treatment paradigm, the mortality rate due to AKI is still as high as 80% in ICU patients [5], [6]. It has been shown that nephrotoxicity alone contributes to large percentages of in-hospital AKI patients.

AKI results from a nephrotoxic or obstructive insult to renal tissue from ischemia reperfusion and tubulo-interstitial inflammation [7], [8]. It is usually diagnosed by increases in serum creatinine or blood urea nitrogen. Different biomarkers viz. interleukin-18 (IL-18), kidney injury molecule-1 (KIM-1), and neutrophil gelatinase-associated lipocalin (NGAL) have been used for the early diagnosis of AKI [9]. Raised levels of pro-inflammatory cytokine in plasma envisage mortality in patients with AKI [10]. Inflammation has now been believed to be a major player in its pathology [11], [12] and as a result, anti-inflammatory response forms the important part of the reno-protective therapies for AKI. Besides inflammation, apoptosis and necrosis are the main pathological changes that occur at the cellular levels but the molecular mechanisms underlying these changes require more comprehensive understanding [13]–[15].

Over the years the role of inflammatory transcription factors, specifically NF-kB family members in pathophysiology of AKI has surfaced [13]–[20]. Accordingly it has been demonstrated that NF-kB family members consists of a group of five structurally related evolutionarily conserved proteins RelA/p65, RelB, c-rel, NF-kB1/p50 and NF-kB2/p52. All the members of this family contain a similar 300 amino acid long domain called Rel homology (RH) domain. These transcription factors form homo- or hetero-dimers to regulate a diverse number of genes by binding to its target sequence on DNA known as the κB site. NF-kB family of transcription factors have been ascribed in various forms of human and experimental kidney injury, where these regulate the expression of various inflammatory genes through such sites [21], [22].

A wide range of kidney injury related stimuli activate NF-kB, including growth factors, cytokines, damage associate molecules, Nod and Toll like receptors, genotoxic stress, immune mediators and mechanical stress [23], [24]. Initial reports suggested a dual regulatory role of NF-kB in a single cell during pro-apoptosis as well as anti-apoptosis [25], [26]. However, despite the knowledge about essential role of NF-kB as well as its associated targets in progression of toxic induced AKI [13]–[20] yet both the early diagnosis and the therapeutics have largely remained the cause of concern. This has necessitated a more rigorous looking at the proinflammtory molecules as well as the associated genes and targets for delineating the events associated with AKI.

The protein p53 is a tumor suppressor protein that primarily responds to DNA damage and cellular stress by interrupting the cell cycle and by stimulating apoptosis [27], [28]. Recent experimental findings show an important role of p53 in regulation of inflammation during ischemic reperfusion injury. Accordingly, an acute pharmacological and genetic absence of p53 brings about a protective role during kidney injury [29]–[31]. Accumulating evidences indicated that p53 also have an κB site at its promoter region suggesting NF-kB binding and also its potential to regulate p53 gene expression [32], [33]. Based on these observations it seems possible that NF-kB and p53 play a crucial role in inflammation and apoptosis, but their importance with respect to AKI remains largely unknown. The present study was thus, carried out to determine the significance of the expression of NF-kB dependent process including expression of p53 in an experimental mouse model of FA AKI. Employing inhibitory studies in this mouse model, the observations support the notion that inhibiting NF-kB expression and associated molecules like p53 indeed ameliorated progression of FA – AKI.

## Materials and Methods

### Ethics Statement

The experimental protocols used in our study were approved by the Institutional Animal Ethics Committee (IAEC) (Approval ID: 49/1999/CPCSEA) of Panjab University, Chandigarh, India and performed in accordance with the guidelines of Committee for the Purpose of Control and Supervision of Experiments on Animals (CPCSEA), Government of India. All efforts were made to minimize the suffering of animals.

### Animals and Drug Treatment

Four to six week old male BALB/c mice weighing 25–35gms, were used and maintained under standard pellet diet and free access to water *ad libitum*. FA was dissolved in 0.2 ml of 0.3 mM sodium bicarbonate (NaHCO3) and administered intraperitonially at a concentration of 250 mg/kg/wt. Control animals received same volume of sodium bicarbonate (0.3 mM). Two hour before FA administration, pyrrolidine dithio-carbamate ammonium (PDTC) (Sigma- Aldrich) (200 mg/kg/wt. in normal saline) was injected intraperitonially.

Before sacrificing, the mice were anesthetized with intraperitoneal injection of sodium pentobarbital (50 mg/kg/wt) and blood was collected from animals by retro-orbital blood collection method for the evaluation of renal function. Animals were then euthanized by cervical dislocation and one of the kidney was fixed in 10% formalin for histological analysis. Half of second kidney was stored in TRIzol for RNA isolation and remaining half kidney was harvested for biochemical analysis.

### Experimental Design

Experiments were performed in two separate stages; stage-1 consisted of time response studies in which animals were divided into two groups, control and FA treated group. The control group were administered 0.2 ml of 0.3 mM NaHCO_3_. Group treated with FA was further divided into three sub-groups i.e. subgroup-A, -B and -C. The animals in these subgroups were administered intraperitoneal injection of FA (250 mg/kg/wt) in vehicle (0.2 ml of 0.3 mMNaHCO_3_) for a period of 6, 12 and 24 hr, respectively. To highlight the expression of NF-kB in FA AKI, PDTC was given two hours prior to FA administration. The stage-2 experimental animals were divided into four groups Control, PDTC, FA, and FA+PDTC. Mice were sacrificed at two time points (i.e. 12 hr and 48 hr) after FA administration.

### Renal Function Tests

Animals were sacrificed and blood was collected from each group under treatment. Serum was prepared for analyzing renal functioning, blood urea nitrogen (BUN) and creatinine, expressed as milligrams per 100 ml (mg/dl), levels were analyzed spectrophotometrically by modified Berthelot method using commercially available kit (Reckon Diagnostics Pvt. Ltd, Baroda, INDIA).

### Microscopic Assessment

For histo-pathological studies renal tissues were fixed in 4% paraformaldehyde solution. Fixed tissues were then dehydrated in a graded series of ethanol solutions (30%, 50%, 70%, 90% and 100%) and embedded in paraffin. Haematoxylin & Eosin (H & E) and Periodic acid Schiff (PAS) staining were performed on 5 µm thick sections. Semiquantitative evaluation of tubular damage was briefly estimated in 6 to 8 high power fields (HPF) (×400) per section. Total histological score (THS) were measured on the basis of tubular degeneration (TD), tubular necrosis and tubulointerstitial inflammation (TIN) and calculated with formula, THS: TD/2+TN+TIN/2 with scoring system (normal THS = 0–2, mild THS = 2–5, moderate THS = 5–8, severe THS >8) [74]. For transmission electron microscopy (TEM) 1 mm tissue were fixed in 4% paraformaldehyde and 2.5% glutaraldehyde in 0.1 M phosphate buffer (pH 7.4) for at least 26 hrs. Post fixation was done in osmium tetroxide in 0.1 M phosphate buffer followed by dehydration with graded series of ethanol. Tissues were than embedded in beam capsules than 0.5–1.0 µm thin sections were made and observed sections under microscope [75], [76]. Sections were than heated in 60°C oven for 48 hours. Alterations in cell organelles following FA AKI were analyzed using transmission electron microscopy (TEM).

### Gene Regulatory Studies

For *in vivo* expression analysis, total RNA was extracted according to the manufacturer's instructions from renal tissue of the respective animal in each groups/subgroup using TRIzol reagent (Invitrogen). The samples were then treated with DNase I using DNA-free kit (Ambion, USA) followed by reverse transcription and amplification of the cDNA employing specific primers (Table 1). The amplified PCR products were then analyzed and compared with the standard markers following electrophoresis on a 2% agarose gel containing ethidium bromide (0.5 µg/ml).

**Table 1:** Mouse mRNA primers for RT-PCR.

Name	Orientation	Sequence	Product Size
P65	Forward	GGCCTCATCCACATGAACTT	245
	Reverse	CACTGTCACCTGGAAGCAGA	
*NF-kB2*	Forward	ACCTTTGCTGGAAACACACC	201
	Reverse	ATGGCCTCGGAAGTTTCTTT	
*p53*	Forward	GATGACTGCCATGGAGGAGT	664
	Reverse	CTCGGGTGGCTCATAAGGTA	
*Bax*	Forward	GGAGACACCTGAGCTGACCT	461
	Reverse	CTCAGCCCATCTTCTTCCAG	
*TNFα*	Forward	ACCACCATCAAGGACTCAAA	395
	Reverse	AAAAGAGGAGGCAACAAGGT	
*GAPDH*	Forward	TCTTGGGCTACACTGAGGAC	150
	Reverse	TGTTGCTGTAGCCGTATTCA	

### Immunohistochemistry

Immunofluorescent examinations for detection of proteins were performed on longitudinally sectioned kidneys using slight modification of previous protocol [77]. Kidney tissues were fixed in 4% paraformaldehyde fixative prepared in phosphate buffered saline (PBS), and were then embedded into paraffin wax having melting temperature 58–60°C. 5 µm tissue sections were prepared and rehydrated with serial alcohol dilutions (100%, 90%, 70%, 50% and 30%) for 2 minutes each. Sections were then blocked with 2% BSA made up in PBS for 30 minutes and antigen retrieval was carried out in 10 mM citrate buffer (pH 6.0) using microwave. Following antigen retrieval the sections were incubated overnight with primary antibodies NF-kB/p65 (C-20), 1∶500 (Sc-372, Santa Cruz Biotechnology); p53 (FL-393) with 1∶500 dilution (Sc-584, Santa Cruz Biotechnology) at 4°C in a humidified chamber. Slides were then washed with PBS and incubated with goat anti-rabbit IgG FITC conjugate affinity-purified secondary antibody (Merck Biosciences) for 2 hr. Finally sections were incubated with propidium iodide (BD Biosciences) to visualize the nuclei (red) and slides were viewed using Nikon Eclipse 80i microscope.

### Oxidative Stress Analysis

After euthanizing, kidneys from 12 hr FA treated animals were removed and rinsed in ice-cold saline. Renal tissue was minced very finely and 10% (w/v) homogenate were prepared in ice cold 0.1 M phosphate buffer, pH 7.4. Standard biochemical assays were used for estimating activities of catalase [79], superoxide dismutase [80], and the levels of lipid peroxidation (LPO) indices measured as malondialdehyde formation [81], generation of reactive oxygen species (ROS) [82] the contents of reduced and oxidized glutathione (GSH & GSSG) [83].

### Statistical Analysis

Data presented here were expressed as the mean ±SEM. Statistical analysis were performed using one way ANOVA from Graph Pad Prism 3.0 (Graph Pad Software, Inc., San Diego, CA). P values <0.05 were considered to be statistically significant.

## Results

### Serum Biochemistry

**Figure 1:**
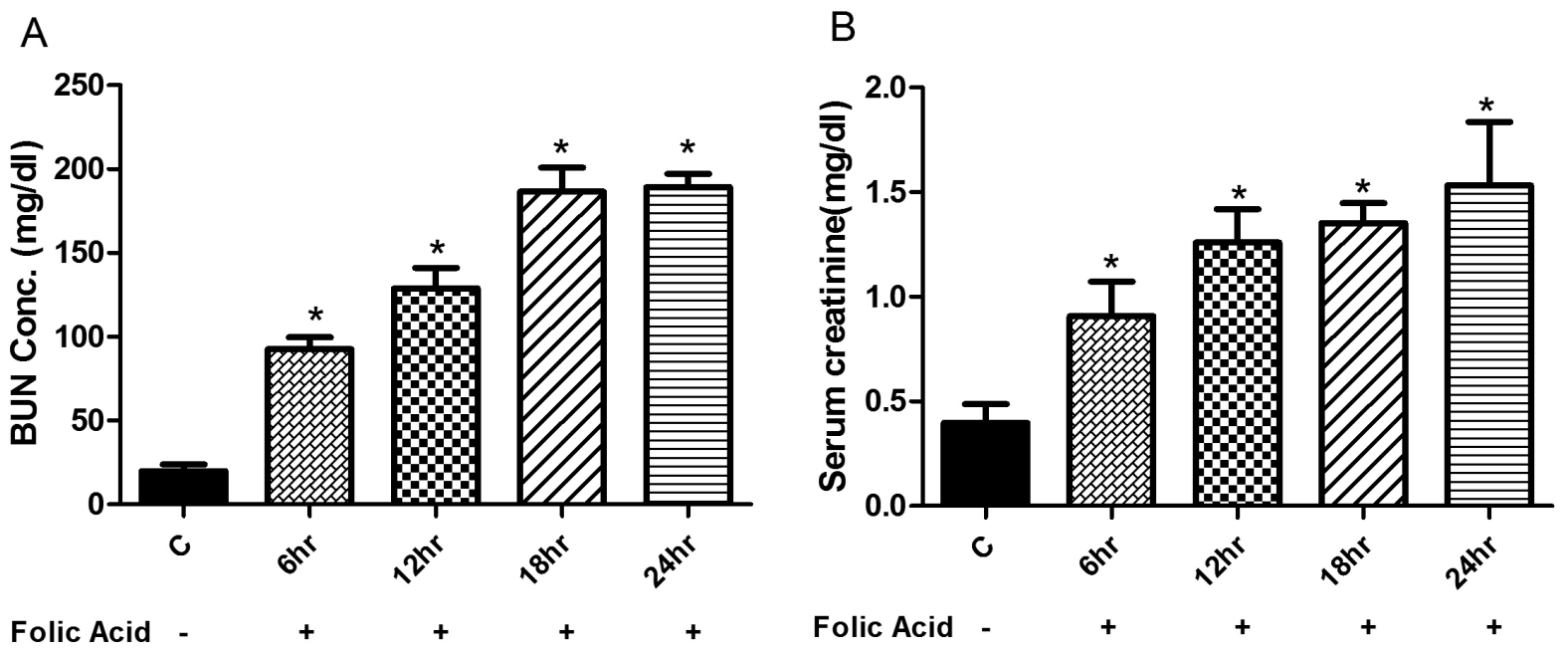
Renal functional response during FA induced injury. BUN and serum creatinine levels were measured at times from 6 to 24 hr after single i.p. injection of FA. Data are expressed as mean ±SEM. Significant differences from FA treatment groups from control group are indicated at *p<0.05.

To establish FA AKI, mice were injected intraperitoneally with a single dose of FA (250 mg/kg/wt) dissolved in 0.3 mM sodium bicarbonate solution. Blood was collected at different time points for measuring serum creatinine and BUN levels. As shown in Fig. 1A, the serum BUN levels initially increased to approximately 4 folds within the first 6 hr and reached to ∼9 folds by 18 hr and were maintained to this high level till 24 hr. Likewise, the level of serum creatinine were found to be elevated to ∼2 folds within first 6 hr and reached to about 3 folds within 18 hr and continued to stay such a high level till 24 hrs post FA treatment (Fig. 1B), suggesting a severe FA induced nephrotoxicity.

### Histopathological Analysis

The biochemical alterations were simultaneously reflected by changes in the renal tissue membrane architecture. With respect to control groups, the light microscopy of H & E stained tissues in the FA treated groups at any time point, demonstrated extensive tubular damage in cortex and medullary regions of kidney. Fig. 2.1A shows control kidney cortex area showing intact tubular architecture. Fig. 2.1B, C and D are the FA treatment groups at different time period (6 hr, 12 hr and 24 hr) respectively. Extensive tubular necrosis is clearly visible in FA treatment groups (arrow). Tubular dilations with flattened epithelium was seen in all groups, moreover individual tubular cells were detached from their membrane leaving denuded thin basement membrane (arrow head). Similarly, medullar region of kidney also shows prominent changes as compared to normal control group (Fig. 2.2A). Extensive tubular necrosis and tubular dilation with flattened epithelium was also observed in all FA treatment groups at varying time intervals viz. 6, 12 and 24 hrs in Figs. 2.2B, 2.2C and 2.2D respectively.

**Figure 2:**
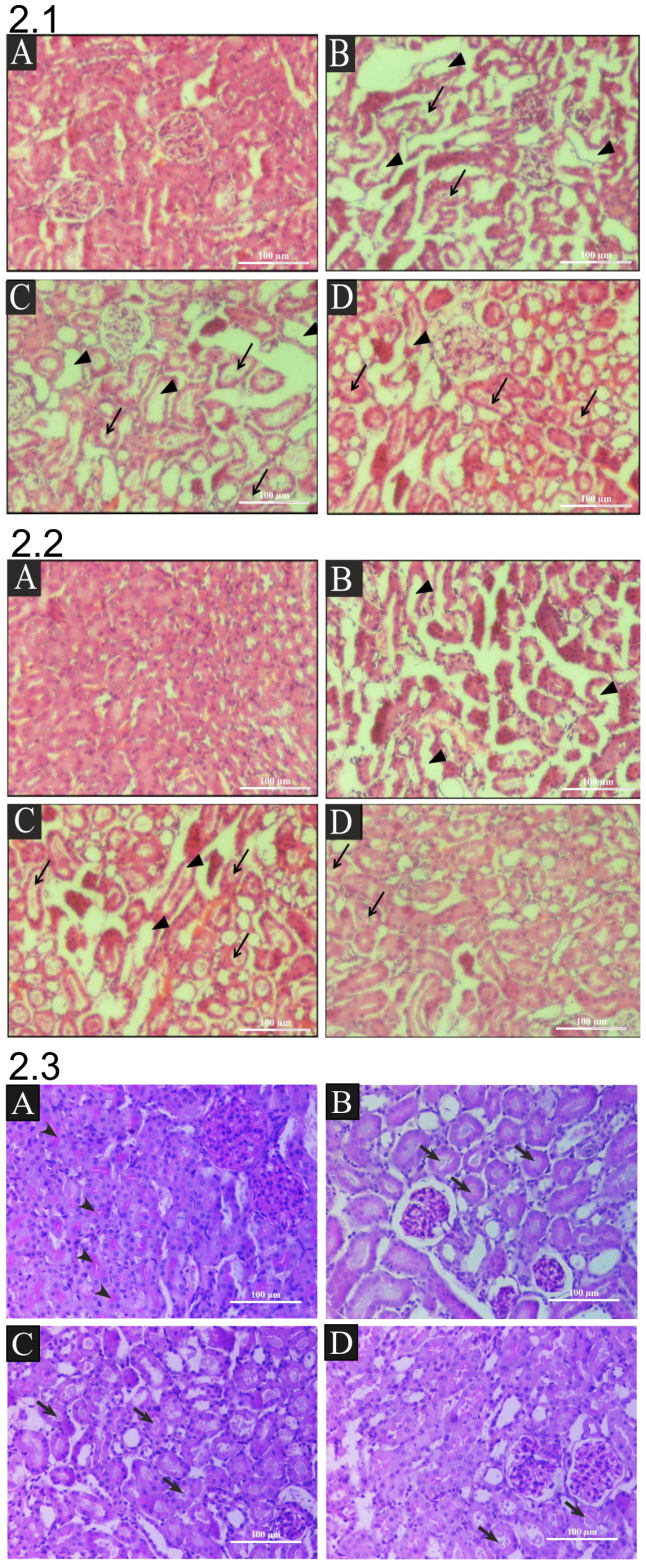
Histological Changes following FA induced injury in mice, 2.1) H & E staining of cortex shows, extensive tubular necrosis (arrow), tubular dilations with flattened epithelium (arrow heads) in all groups shown at original magnification of X200. (A) Control, (B) 6 hr post FA treatment, (C) 12 hr post FA treatment, (D) 24 hr post FA treatment, **2.2) H & E staining of medulla** shows, extensive tubular cell loss following luminal cast formation (arrow), tubular dilations with flattened epithelium (arrow heads) in all groups shown at original magnification of X200 (A) Control, (B) 6 hr post FA treatment, (C) 12 hr post FA treatment, (D) 24 hr post FA treatment, **2.3) Periodic staining (PAS) of renal sections** shows, intact brush boarder in control (arrow head). Extensive loss of brush border membrane and Luminal cast (arrow) were observed in all FA treated groups at original magnification of x200, (A) Control, (B) 6 hr post FA treatment, (D) 12 hr post FA treatment, (D) 24 hr post FA treatment. Scale bar in each case: 100 µm.

Periodic acid staining (PAS) is generally used for the staining of brush boarder membrane (arrow head) within the cells of proximal tubule. PAS of serial sections of control group (Fig. 2.3A) clearly revealed the presence of intact brush boarder membrane on the luminal side of proximal tubule. Whereas, in all FA treatment groups i.e. 6 hr, 12 hr and 24 hr (Fig. 2.3B, 2.3C and 2.3D) extensive loss of brush border membrane and luminal cast deposition (arrow) in the tubular lumen was clearly detectable. Thus, based on the total histological score depicted in S7 Fig., it is obvious that folic acid treatment is quick to induce severe architectural changes indicative of AKI. As can be seen within 6 hrs of its treatment the THS already crossed a values (>8) indicative of level of severity (as elaborated in Method section). This value continue to increase further and peaked 12 hrs (∼25) & remained at such high level 24 hrs post folic acid treatment (S7 Fig.).

**Figure 3:**
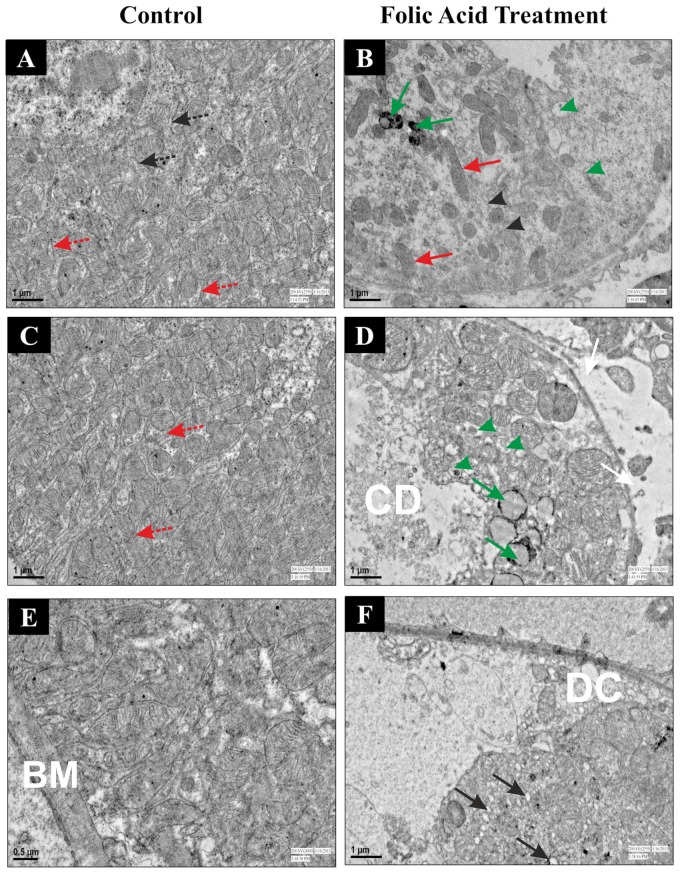
Transmission electron microscopic analysis of renal cortex after FA induced injury in mice. Control (A, C and E) and FA treated (B, D and F) were sacrificed 12 hr after FA induced renal injury. Kidney sections from control and FA treatment were analyzed by TEM. Intact ER cisternae profiles were observed in control group (Fig. 3A, Black dotted arrow). Small degraded fragments of ER were observed in the cytoplasm (Fig. 3B, Black arrow heads) of FA treated group. Dotted red arrow indicates presence of intact basal infoldings in the control group (Fig. 3A). Red arrows demonstrate structure alteration with decreased in number of mitochondria in FA treated group (Fig. 3B). Presence of granular materials (Fig. 3B, Green arrow) and vacuole (Fig. 3F, Black arrow) in cytoplasm, ruptured basement membrane (Fig. 3D, White arrow), partially filled cytoplasmic debris in lumen of tubular cells (Fig. 3D, CD) and detachment of cells from tubular basement membrane (Fig. 3F, DC) were clearly observed in FA treated group.

These results were further verified by transmission electron microscopy (Fig. 3) of the kidney. TEM of tubular cells of control mice is characterized by intact basement membrane (BM) and basal infoldings (red dotted arrow). Mitochondria were abundantly present and have round or elongated shape. A well-defined arrangement of mitochondrial cisternae was clearly observed in control group (Fig. 3A, 3C and 3E). Cisternae profiles of ER were located between the mitochondria near to the nucleus (Fig. 3A, black dotted arrow). FA treated group shows disrupted basal infoldings with ruptured basement membrane (Fig. 3D white arrows), decreased mitochondria with regression of cisternae (Fig. 3B, red arrows), and presence of granular materials in renal tubular cells was clearly noticed (Fig. 3B and 3D, green arrow). ER is not distinguishably observed in between the mitochondria as observed in the control group but small degraded fragments were observed in the cytoplasm (Fig. 3B, black arrow heads) suggesting excessive ER stress. Cytoplasmic debris (CD) in tubular lumen, vacuole formation (black arrow), increased number of lysosomes (green arrow head) and detachment of cells from tubular basement membrane (DC) was clearly visible in FA treated animals (Fig. 3B, 3D, 3F).

### Expression of relA, NF-kB, and p53 in relation to AKI

**Figure 4:**
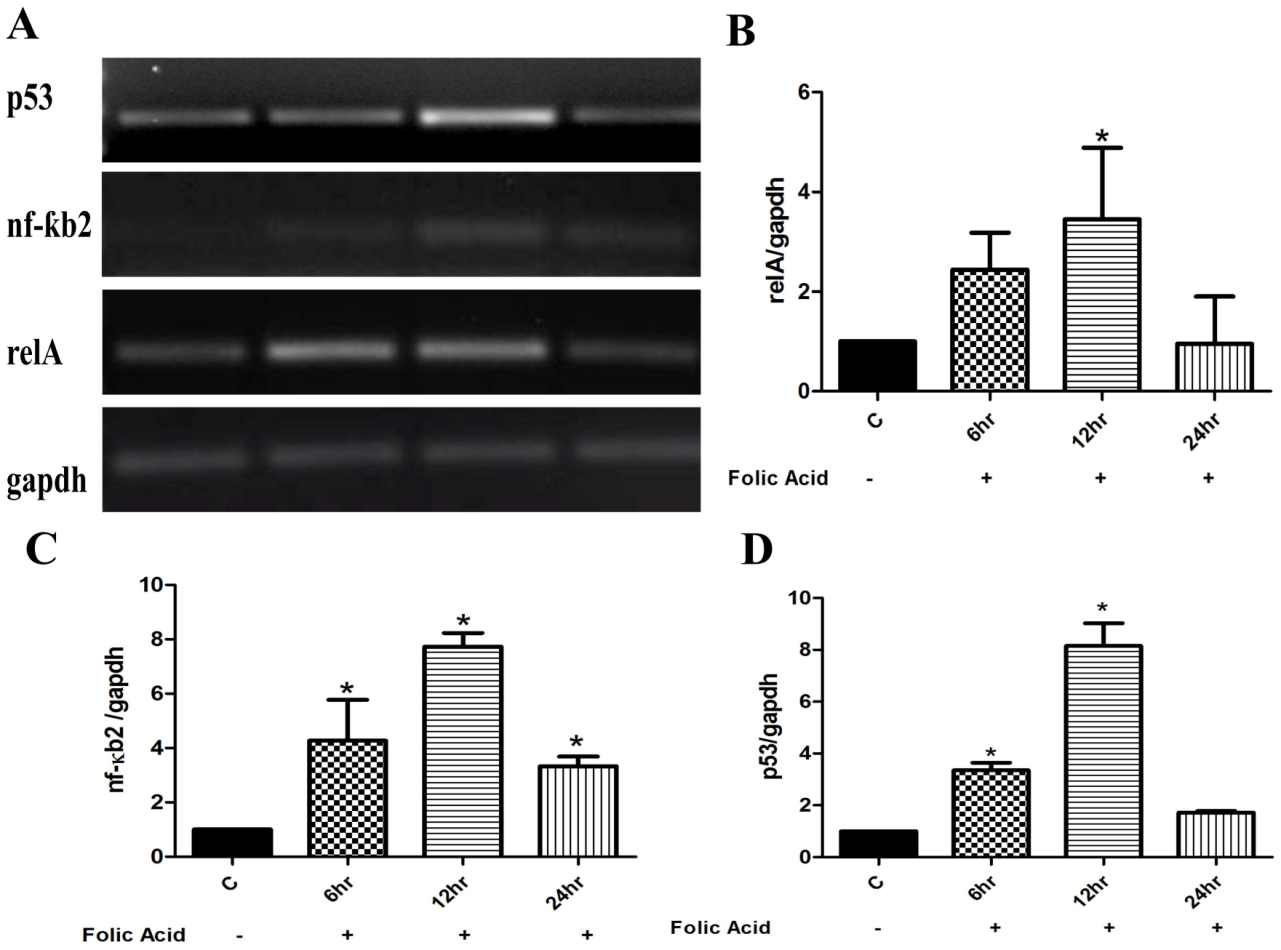
Time-dependent changes in level of renal genes after FA induced injury in mice. Representative gel electrophoresis images of RT-PCR products of control and FA treatment shows the expression of relA (∼4 folds), nf-kb2 (∼8 folds), and p53 (∼8 folds) were maximum at 12 hr of FA injury which further reduced to normal level at 24 hr. The mRNA levels were normalized by the expression of GAPDH in each experiment, and were expressed as % fold change relative to control animals. Data are presented as mean ±SEM (N = 3 animals per group). *P<0.05compared between control and FA treated group.

To examine the renal injury associated with changes in expression of aforementioned genes, RT-PCR analyses were performed to observe the changes in mRNA levels. The electrophoretogram of the amplified RT-PCR products reveled significant changes in the expression of these genes. The level of amplification was quantified by densitometric analysis of PCR products relative to the housekeeping gene, GAPDH. Our results demonstrated an increase in relA mRNA expression (Fig. 4A & 4B) within 6 hr and reaching to highest level with in 12 hr (∼4 folds). However, 24 hr post-treatment of FA, relA expression was observed to be at par with that seen for the control groups (Fig. 4A & 4B). Having observed increase in relA expression, a potential increase in NF-kB2 mRNA levels was expected due their combinatorial regulation of gene expressions. To further analyze congruent increase in NF-kB2 level along with relA, RT-PCR analysis was performed employing primers specific to NF-kB2. As shown in Figs. 4A & 4C, mRNA expression of NF-kB2 was observed to be increased to ∼8 folds at 12 hr time interval while declined at 24hr post treatment. These results, therefore, verified that expression levels of relA and NF-kB2 indeed compatibly work in concert with each other in regulating processes during AKI. Likewise, the expression of p53 was also found to be enhanced to its maximum levels at 12 hr (> 8 fold) and then decline during 24 hr time interval (Fig. 4A and 4D).

### NF-kB dependent expression of p53 in folic acid induced renal tissue

**Figure 5:**
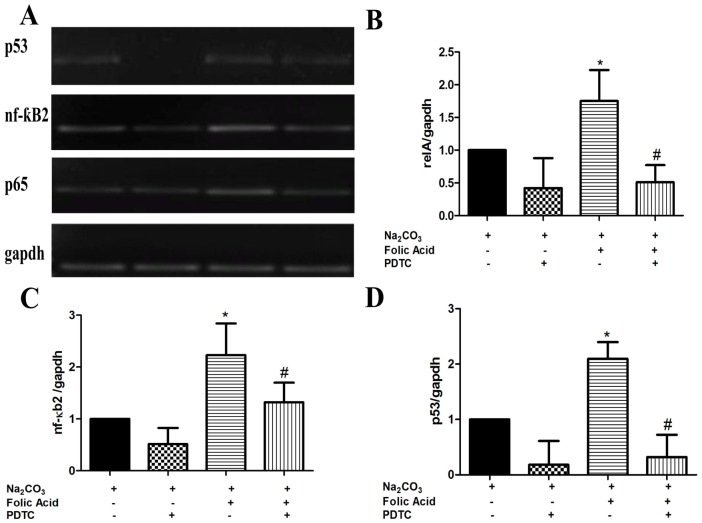
Effects of PDTC on renal genes expression after FA induced injury in mice. The mRNA expression of p65 (B), nf-kb2 (C), and p53 (D), was measured at 12 hours after injection of FA+vehicle or FA+PDTC byRT-PCR. The mRNA levels were normalized by the expression of GAPDH in each experiment, and were expressed as % fold change relative to control animals. Data are presented as mean ±SEM (N = 3 animals per group). *P<0.05 compared between control and FA treated group. #P<0.05 compared between FA treated group and FA+PDTC treated group.

In an attempt to find out whether the regulation of p53 following FA induced AKI is indeed NF-kB dependent, a known inhibitor of NF-kB, PDTC was exploited. Mice were administered PDTC 2 hr before FA administration and mRNA expression was analyzed after 12 hr of FA induced injury. As shown in Figs. 5A, 5B & 5C, increased mRNA expressions of relA and NF-kB2 in FA induced mice kidney were significantly reduced in PDTC treated groups. Along with reduced levels of NF-kB and relA, the level of expression of p53 was also observed to be decreased in kidneys of PDTC treated mice in comparison to FA induced mice renal tissue (Fig. 5A & 5D). These observations therefore, strongly suggested that reduction in NF-kB2 and relA along with p53, points towards their correlative association during pathological changes occurring during FA induced AKI.

**Figure 6:**
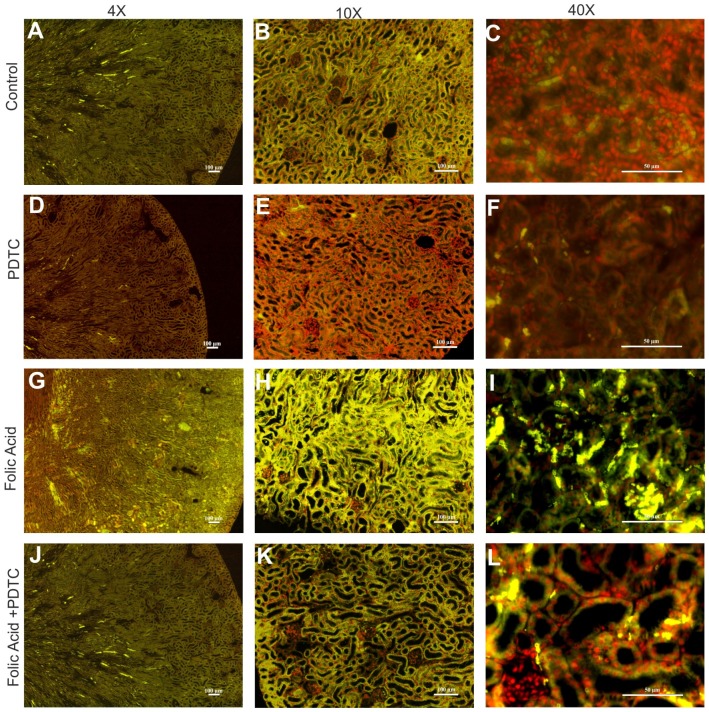
Renal NF-kB protein expression in FA induced injury in mice. Representative images of NF-kB immunostaining (green: FITC and red: Propidium iodide) showing decrease in protein expression in PDTC treated mouse kidney at 48 hr after FA treatment (A–L). n = 4; Magnification: 40X, 100X, and 400X with scale bar: 100 µm, 100 µm and 50 µm respectively.

**Figure 7:**
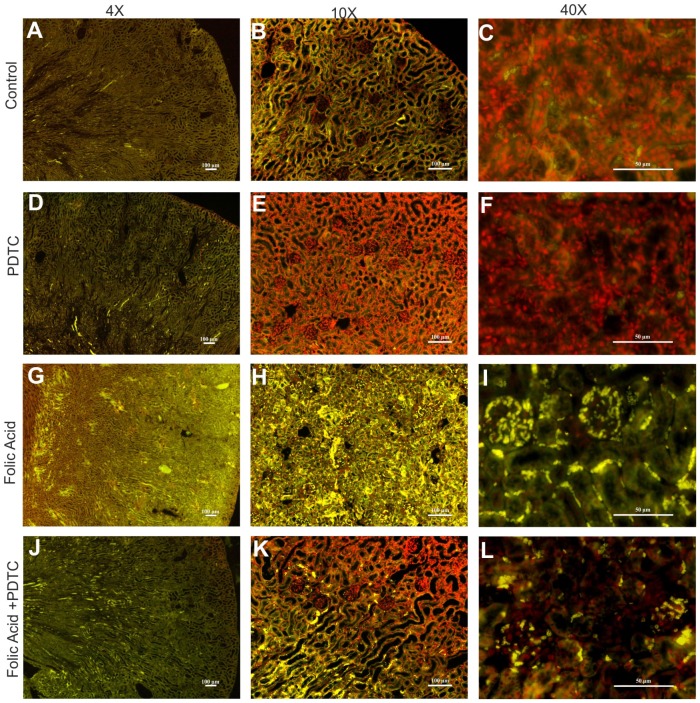
Renal p53 expression in FA induced injury in mice. Representative images of p53 immunostaining (green: FITC and red: Propidium iodide) showing decrease in protein expression in PDTC treated mouse kidney at 48 hr after FA treatment (A–L). n = 4; Magnification: 40X, 100X, and 400X with scale bar: 100 µm, 100 µm and 50 µm respectively.

Immunofluorescence of paraffin embedded renal tissue from control and FA induced AKI showed increased accumulation of NF-kB (Fig. 6, Green) and p53 (Fig. 7, Green) protein levels in renal tissue in FA treated mice as compared to the control mice. The elevated expressions of NF-kB and p53 protein after FA induced renal injury was decreased when treated with PDTC, thus, demonstrating that expression of p53 indeed depends on NF-kB (Fig. 6 & Fig. 7). Besides these protein's accumulation within renal tissue, it was also observed that FA treatment rendered both NF-kB & p53 nuclear bound (S1 & S2 Figs., Panel Merged) suggesting their activation during FA induced AKI. Restitution of these proteins back to cytoplasm following PTDC pretreatment (Panel Localization, S1 & S2 Figs.) additionally reiterated that NF-kB functions upstream in regulating p53 expression during FA induced AKI. Hence, in consequence, the functional importance of this effect of NF-kB in FA induced AKI was evinced by a significant enhancement (∼6.5 fold) in the expression of this cytokine, (S3 Fig.) as well as generation of pro-oxidant state (S4 & S5 Fig.) and upregulation of pro-apoptotic, Bax gene expression (S6 Fig.). The notion that this increase was NF-kB dependent was reassured through inhibition of TNFα expression brought about by PTDC pretreatment in FA treated animals (S3 Fig.). Additionally, PTDC pretreatment abrogated the FA induced oxidative stress in FA induced AKI model. (S4 & S5 Figs.) and lowered the expression of Bax gene (S6 Fig.).

### Suppression of Folic acid-induced renal injury by PDTC

**Figure 8:**
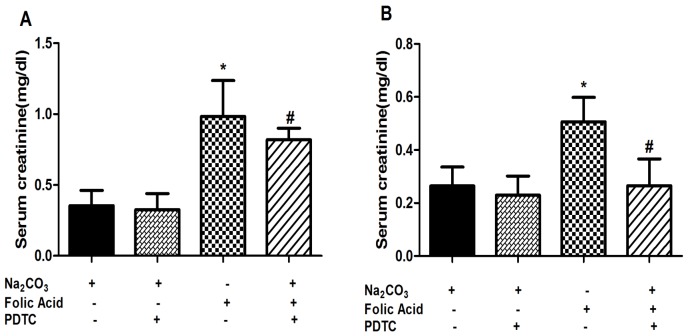
Recovery of renal function on treatment with PDTC. The level of serum creatinine was measured at 12 hr (A) and 48 hr (B) after single i.p. injection of FA. PDTC was administered 2 hr before FA treatment. Data are presented as mean ±SEM (N = 6 animals per group). *P<0.05 compared between control and FA treated groups. #P<0.05 compared between FA treated group and FA+PDTC treated group. Magnification: 400X, scale bar: 100 µm.

**Figure 9:**
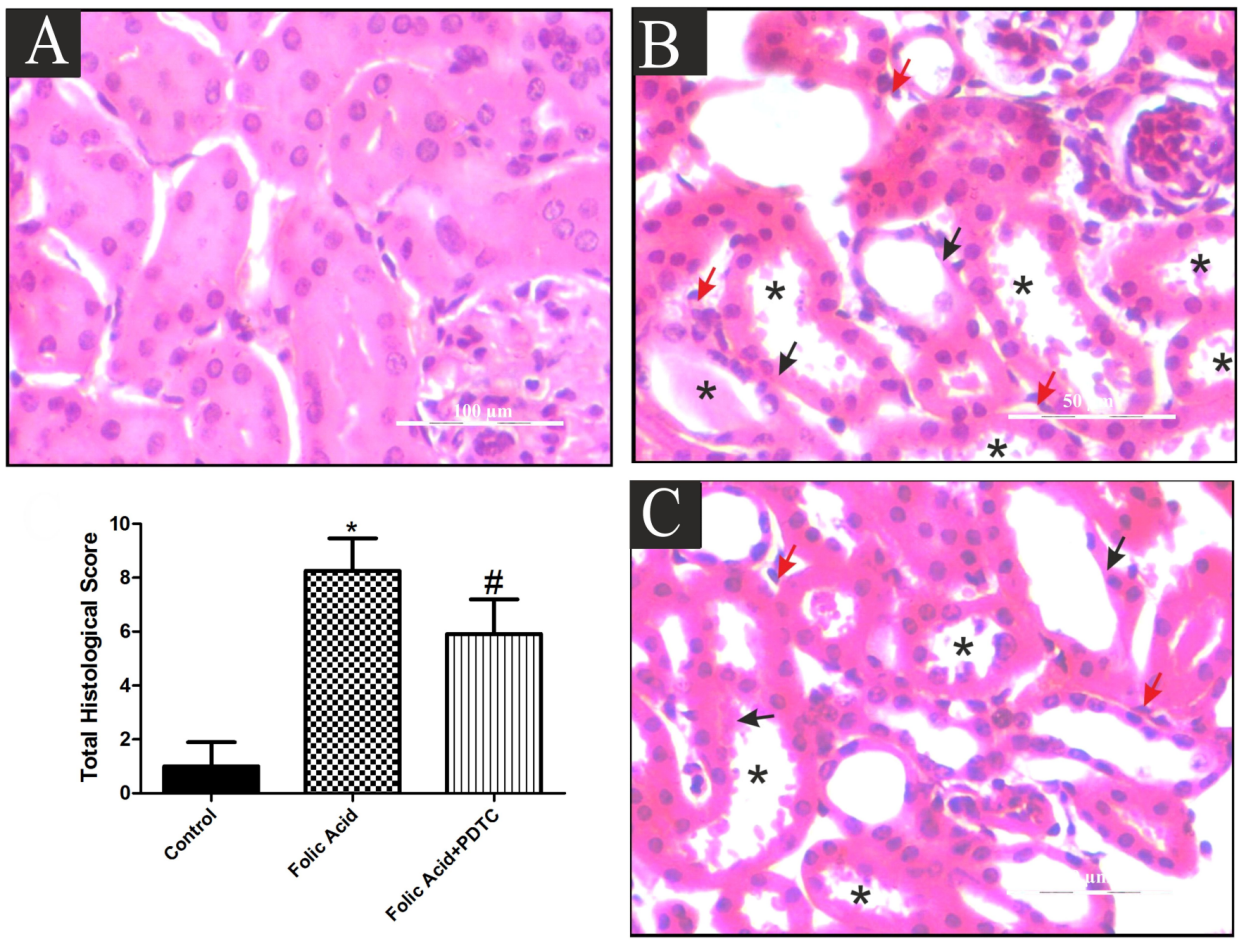
Transient inhibition of NF-kB ameliorates kidney injury after FA induced injury in mice. Representative micrographs of renal tissue in Control (A), FA (B) and FA+PDTC (C) in mice at 48 hr post renal injury are shown along with total histological score calculated as tubular degeneration (Black arrow), tubular necrosis and cast formation (asterisks) and tubulointerstitial inflammation (Red arrow) in the enlarged boxed areas. Semiquantitative assessment of renal injury was presented as number of damaged tubule per total cross-sectional area of renal tissue from respective group of animals. *P<0.05 (n = 3).

After examining the role of NF-kB in FA induced nephrotoxicity, effects of PDTC on FA AKI mice model was examined in order to determine its therapeutic effects. Immuno-histological findings showed that PDTC treatment reduced the accumulated levels of NF-kB in FA treated mice. Having observed role of NF-kB in these AKI mice models, PDTC treatment also showed inhibition in progression of AKI of the FA treated mice. Serum creatinine levels were decreased from 0.985 mg/dl in FA treated animals to 0.819 mg/dl in FA+PDTC treated animals at 12 hr (Fig. 8A). A higher reduction in levels of serum creatinine were observed at 48 hr where its level decreased from 0.506 mg/dl in FA treated animals to 0.289 mg/dl in FA+PDTC treated animals (Fig. 8B). Moreover H & E staining of renal tissue also showed reduction in tubular injury in FA+PDTC treated animals compared to FA treated animals (Fig. 9). The total histological score (THS) measured on the basis of tubular degeneration (TD), tubular necrosis and tubulointerstitial inflammation (TIN) again reiterated reduced tubular injury in FA+PDTC treated mice (Fig. 9).

## Discussion

The acute kidney injury (AKI) is a highly complex disorder. The variability in the disease affliction further adds to the intricacy associated with this disease. Over the years the role of inflammation has been the focus of attention in understanding the disease pathogenesis. Experimental studies in AKI have utilized different models to demonstrate an association with a strong inflammatory reaction. It is believed that during AKI, the preliminary reaction starts with morphological and/or functional changes in vascular endothelial cells and/or in tubular epithelium [34]–[36] leading to renal tissue injury. The consequent generation of inflammatory mediators like IFNγ, IL-2, IL10, GM-CSF, TGF-β, CXCL1, IL-6, MIP-2, and MCP-1 by tubular and endothelial cells are thought to play an important role in AKI pathophysiology [37]–[41]. Along with these factors oxidative stress has also been demonstrated to be an important factor in the pathogenesis of AKI [42], [43]. Thus a close relationship among these components is possible. Previous observations from our laboratory demonstrated that acute administration of FA leads to the generation of oxidative stress and altered membrane architecture responsible for FA AKI [44]. Extending earlier study further, we now show that NF-kB, also a transcription factor plays an important role in regulation of FA AKI. A time responsive change in the mRNA as well as protein expression of NF-kB in FA AKI shows its role during renal injury. Parallel to NF-kB expression, similar time responsive changes in the level of p53 mRNA as well as protein suggested a correlation between them. Chemical inhibition of NF-kB by PDTC simultaneously also lowered the expression of p53 mRNA as well as protein leading to early restoration of renal function from FA AKI. A fine tuning of these transcription factors thus, opens up an approach which can be exploited both to study the molecular mechanisms associated with AKI and for possible therapeutic intervention. The fact that two processes i.e. oxidative stress and inflammation are closely linked to disease affliction in renal tissue [45]–[48], the very possibility of delineating the role of inflammation in AKI thus became highly relevant. Such close relationship has indeed been observed in the present study. As depicted in supplementary information (S2 & S3 Figs.), the FA treatment produced proxidant state in the renal tissue as observed by decreased levels of antioxidant enzymes activities viz. catalase & superoxide dismutase (S2A & B Fig.) while increased formation of lipid peroxide indices as depicted by malondialdeyhde (MDA) formation (S2C Fig.) and increased generation of reactive oxygen species (ROS) (S2D Fig.) with simultaneous reduction in the levels of reduced glutathione (S3A–C Fig.). Treatment of the animals with NF-kB inhibitor PDTC rendered protection against FA induced oxidative stress (S2 & S3 Figs.). These observations thus reiterate the close association of the two pathways i.e oxidative stress and inflammation in the etiology of FA induced AKI.

Many of the cytokines, chemokine's and other cell cycle regulatory elements have been demonstrated to be involved in the regulation of inflammation during kidney injury [37]–[41], [49]. These inflammatory cytokines result in activation of many downstream signaling pathways involving NF-kB dependent activation of numerous target genes [23], [50], [51]. Simultaneously, cellular apoptosis has now been observed to be coupled to inflammatory response. Both these processes are now being viewed as tightly coupled processes and are tuned to be regulated through a balance among inflammatory cytokines pro and anti-apoptotic factors in which transcription factor p53 is known to play a crucial regulatory role [52], [53]. In this regard, observations of this *in vivo* study indeed lend a support to such a relationship. Observed higher expression of cytokine, *TNFα*, as well as a proapoptotic gene target of *p53* [78], the *Bax*, by folic acid treatment and downregulation following PTDC pretreatment provided strong evidence for NF-kB dependency on inflammatory and the apoptotic following FA induced AKI, (S4 Fig.). The results, thus, provided sufficient evidence to show NF-kB and p53 interactions are important in pathogenesis of FA AKI.

NF-kB family of transcription factors plays a key role in induction and regulation of inflammation in many pathological conditions both through a classical as well as alternate pathways [54]–[56]. Both these pathways also form the part of events enroute AKI, as could be seen by upregulation of RelA and NFκB2, following FA treatment in time dependent manner. Accumulating evidences have shown that overexpression of RelA gene potentiates cell death depending upon the type of stimulus [57]–[60].

The present study also demonstrated that besides NF-kB, the renal expression of p53 is simultaneously up regulated in a similar time responsive manner in FA treated mice. A recent observation on renal tissue toxicity, also reported that both NF-kB and p53 were up regulated in proximal tubular cells confirming their interaction during regulation of tissue injury [61]. Indeed, NF-kB transcription factors can act both as activators and repressors of transcription of different target genes that depend upon its mode of induction by different stimulus [62]. In this context, we hypothesized that the suppression of NF-kB transcription factor by a chemical inhibitor (PDTC) might open an avenue to understand signaling pathway leading to regulation of FA AKI. Our findings show that pre-treatment of PDTC 2 hr before FA, reduced expression levels of RelA and NF-kB2 and this inhibition was escorted by marked increase in renal function showing a decreased serum creatinine levels and also demonstrating histological protection. Surprisingly, pre-treatment with PDTC also decreased the expression of p53. This finding therefore suggested that these two important cell cycle and apoptosis regulatory elements are indeed inter-dependent during progression of AKI. Further lending support to our hypothesis, immuno-fluorescence studies of renal tissue also showed an elevation in the levels of NF-kB and p53 proteins after FA AKI, which were further reduced by pre-treatment of PDTC. Gene regulatory studies on two cancerous cell lines, Saos-2 and RKO cells, report p53-mediated cellular apoptotic death is mediated via NF-kB activation [59] thus supporting our results. Accumulating evidences also show that p53 dependent cessation in cell growth is regulated by its interaction with NF-kB transcription factors [33], [63]. Recent studies on toxicity of indoxyl sulfate on proximal tubule cells showing association with increase in the level of ROS with activation of NF-kB, and p53, also support our present data [64]–[66].

Thus based on the observations of the present study together with the corroborations from our previous observations [44], a collective mechanism for FA AKI emerges out. Accordingly, acute high dose of FA overwhelms the renal tubules leading to pro-oxidant state in the tissue. This forms the second messenger system to activate NF-kB by ensuing inflammation. Inflammation is known to severely affect blood flow to the outer medulla with consequent functional abnormality to tubular function [67]. Thus, the altered architecture as observed following FA AKI as observed in the present study turns out to be the result of multiple events, one that proceeds following FA induced pro-oxidant state together with the consequences of the inflammation & apoptosis following activation of NF-kB. The activated NF-kB, then gets nuclear bound and targets the promoters of various genes including p53 and TNFα as shown in the present study. The down-regulation of such a molecular pathway alongside the restoration of renal function by PTDC offers an interesting notion. It is rather known that activated NF-kB results in mesengial cell activation with consequent renal injury [68], [24]. The p53 generally functions in a beneficial manner and is known to stabilize the genome for handling any stress [69], [70]. However, under the conditions of acute stress, the character of this protein changes from a safeguard protein to a stress builder proteins [71]. In the present study, simultaneous activation of NF-kB and p53 during FA AKI and restoration of both the renal function and expression of these molecules by PDTC suggested a variable response of p53. This certainly point towards a noteworthy observation regarding proapoptotic role of p53 during FA AKI. It may very well be in line with the observations which demonstrated that damage to the spinal cord as seen during the radiation exposure is brought about by p53-dependent apoptosis of oligodendroblasts [72]. The reiteration to this effect was further reassured upon demonstrating that mice deficient in p53 did not suffer any apoptosis as well as reduced prevalence of paralysis following radiation exposure in these cells within the spinal cord. Besides this indirect indication, the observations [27], [73], [28] have also directly demonstrated the involvement of p53 in producing toxicity upon use of a chemotherapeutic agent, cisplatin and hence restraining its use and effectiveness as an anticancer treatment. Further, the observations that cisplatin induced nephrotoxicity was negated in p53-deficient mice reaffirmed that p53 plays a critical role in the sensitivity of the kidney tissue to genotoxic stress. These reports together with the observations of the present study, therefore, lend sufficient credibility to the fact that a rational therapeutic paradigm must be in place to take in account the status of the molecular components specifically, NF-kB and p53. The crosstalk of both these molecular components thus must be assessed so as see if these form the part of a preliminary diagnostic for different disease conditions specifically AKI.

## Supporting Information

S1 Fig **Immunolocalization of NF-kB.** Animals were injected with 250 mg/kg/wt of FA and killed 48 hr later. Some animals were pre-treated with PDTC 2 hr before FA administration. Kidneys were harvested from mice and paraffin-embedded kidney sections were analysed by immune-histochemical staining. Red panel demonstrates PI stained nucleus of the cells and Green panel in the images demonstrate presence of NF-kB (FITC labelled). Merged images demonstrate nuclear localisation of NF-kB and distribution in renal cortex and the localization panel show the magnified area selected (white Square) to pinpoint variation in colour intensity owing to nuclear localization of NF-kB. n = 4; Magnification: 400X, scale bar: 50 µm.
(DOCX)

S2 Fig **Immunolocalization of p53.** Animals were injected with 250 mg/kg/wt of FA and killed 48 hr later. Some animals were pre-treated with PDTC 2 hr before FA administration. Kidneys were harvested from mice and paraffin-embedded kidney sections were analysed by immune-histochemical staining. Red panel demonstrates PI stained nucleus of the cells and Green panel in the images demonstrate presence of p53 (FITC labelled). Merged images demonstrate nuclear localisation of p53 and distribution in renal cortex and the localization panel show the magnified area selected (white Square) to pinpoint variation in colour intensity owing to nuclear localization of p53. n = 4; Magnification: 400X, scale bar: 50 µm.
(DOCX)

S3 Fig **Effects of PDTC on TNF-α gene expression after FA induced injury in mice.** The mRNA expression of TNF-α was measured at 12 hours after injection of FA+ vehicle or PDTC+FA by RT-PCR. The mRNA levels were normalized by the expression of GAPDH and were expressed as % fold change relative to control animals. Data are presented as mean ±SEM (N = 3 animals per group). *P<0.05 compared between control and FA treated group. #P<0.05 compared between FA treated group and FA+PDTC treated group.
(DOCX)

S4 Fig **Effect of PDTC on activity of antioxidant enzymes and ROS and LPO after FA induced injury in mice.** (A) SOD, (B) catalase, (C) ROS and (D) LPO in mouse kidney homogenate of FA treated animals. Data are presented as mean ±SD (N = 6 animals per group). *P<0.05 compared between control and folic acid treated groups. #P<0.05 compared between FA treated group and FA+PDTC treated group.
(DOCX)

S5 Fig **Effect of PDTC on glutathione levels and redox ratio after FA induced injury in mice.** (A) GSH Levels, (B) GSSG levels and (C) redox ratio in mouse kidney homogenate of FA treated animals. Data are presented as mean ±SD (N = 6 animals per group). *P<0.05 compared between control and folic acid treated groups. #P<0.05 compared between FA treated group and FA+PDTC treated group.
(DOCX)

S6 Fig **Effects of PDTC on Pro-apoptotic gene,** ***Bax*** **expression after FA induced injury in mice.** The mRNA expression of Bax, was measured at 12 hrs after injection of FA+ vehicle or PDTC+FA by RT-PCR. The mRNA levels were normalized by the expression of GAPDH and were expressed as % fold change relative to control animals. Data are presented as mean ±SEM (N = 3 animals per group). *P<0.05 compared between control and FA treated group. #P<0.05 compared between FA treated group and FA+PDTC treated group.
(DOCX)

S7 Fig **Tubular injury score in FA induced injury in mice.** Total histological score calculated in Control and FA induced mouse kidney sections at different time point (6, 12 and 24 hrs). Semi-quantitative assessment of renal injury was presented as number of damaged tubule per total cross-sectional area of renal tissue from respective group of animals. Magnification: 200X, *P<0.05 compared between control and FA treated groups. (n = 3).
(DOCX)
