# Supplementary figures and images for: The Restrained Expression of NF-kB in Renal Tissue Ameliorates Folic Acid Induced Acute Kidney Injury in Mice
Source: PLoS One. 2015 Jan 5;10(1):e115947. doi: 10.1371/journal.pone.0115947 (PMC4283964; doi:10.1371/journal.pone.0115947)

**Figure. S-1. Immunolocalization of NF-kB.**


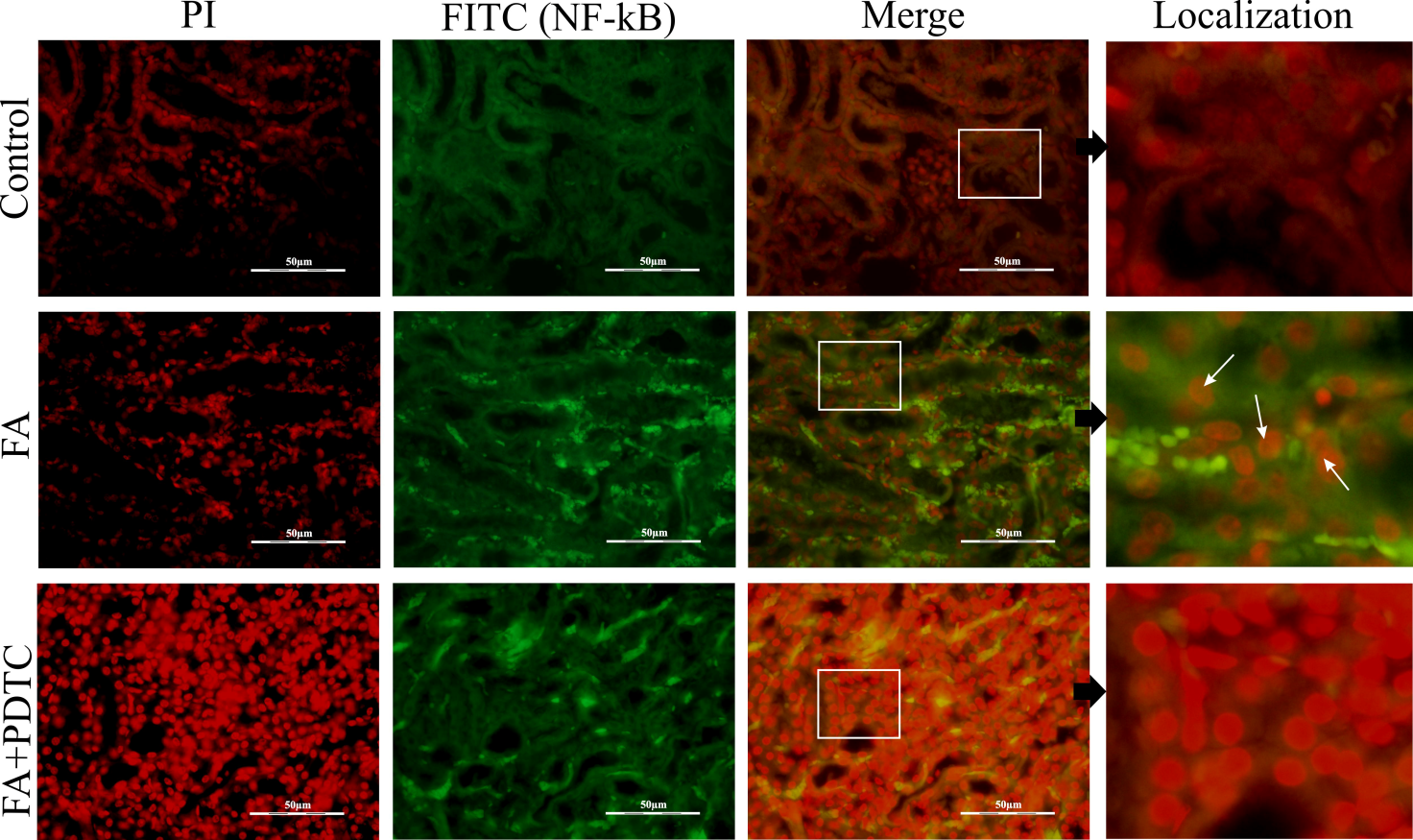

Supplement: S1 Fig — Immunolocalization of NF-kB. Animals were injected with 250 mg/kg/wt of FA and killed 48 hr later. Some animals were pre-treated with PDTC 2 hr before FA administration. Kidneys were harvested from mice and paraffin-embedded kidney sections were analysed by immune-histochemical staining. Red panel demonstrates PI stained nucleus of the cells and Green panel in the images demonstrate presence of NF-kB (FITC labelled). Merged images demonstrate nuclear localisation of NF-kB and distribution in renal cortex and the localization panel show the magnified area selected (white Square) to pinpoint variation in colour intensity owing to nuclear localization of NF-kB. n = 4; Magnification: 400X, scale bar: 50 µm. (DOCX) [file pone.0115947.s001.docx]

**Figure. S-2. Immunolocalization of p53.**


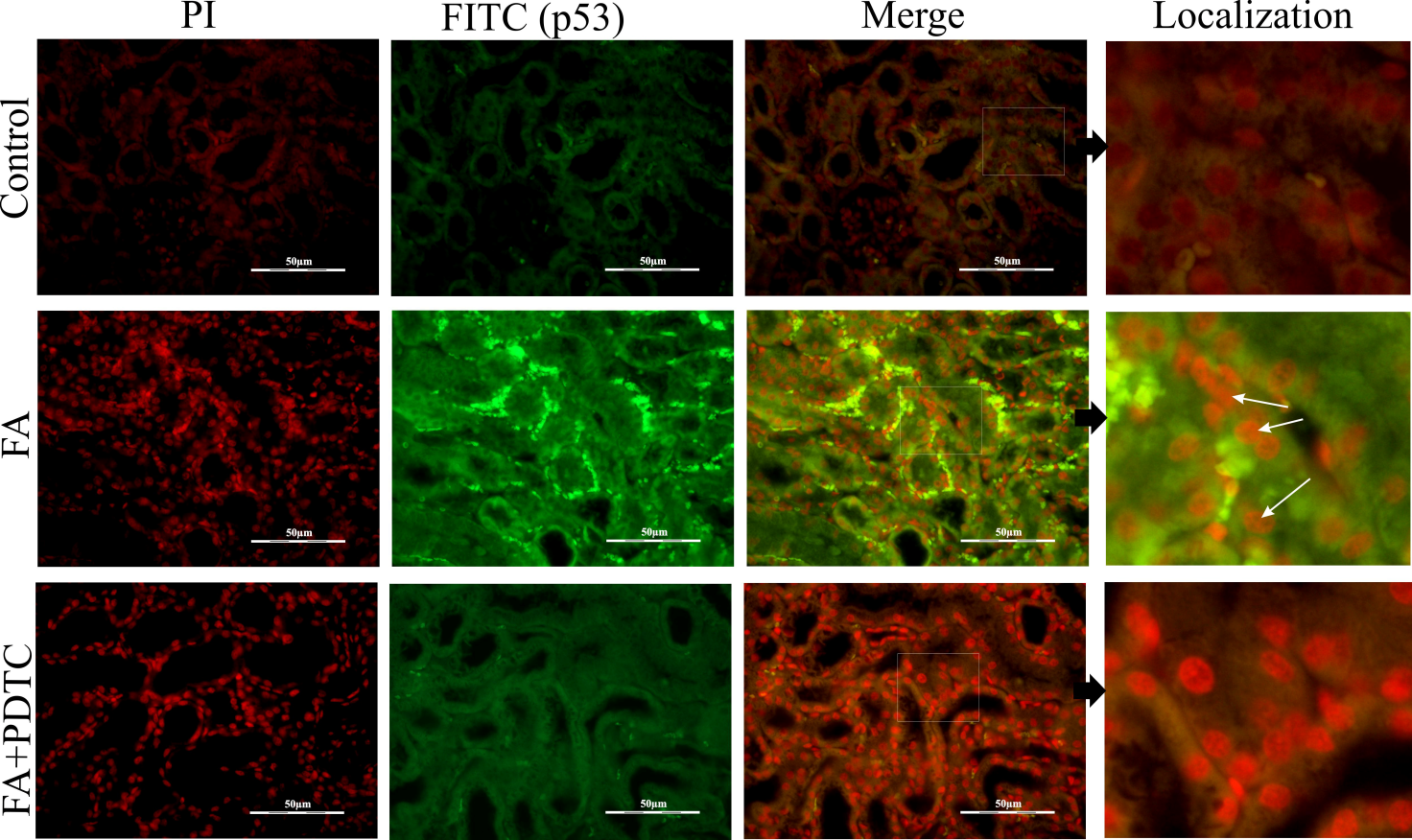

Supplement: S2 Fig — Immunolocalization of p53. Animals were injected with 250 mg/kg/wt of FA and killed 48 hr later. Some animals were pre-treated with PDTC 2 hr before FA administration. Kidneys were harvested from mice and paraffin-embedded kidney sections were analysed by immune-histochemical staining. Red panel demonstrates PI stained nucleus of the cells and Green panel in the images demonstrate presence of p53 (FITC labelled). Merged images demonstrate nuclear localisation of p53 and distribution in renal cortex and the localization panel show the magnified area selected (white Square) to pinpoint variation in colour intensity owing to nuclear localization of p53. n = 4; Magnification: 400X, scale bar: 50 µm. (DOCX) [file pone.0115947.s002.docx]

**Figure. S-3 Effects of PDTC on TNF-α gene expression after FA induced injury in mice.**

**
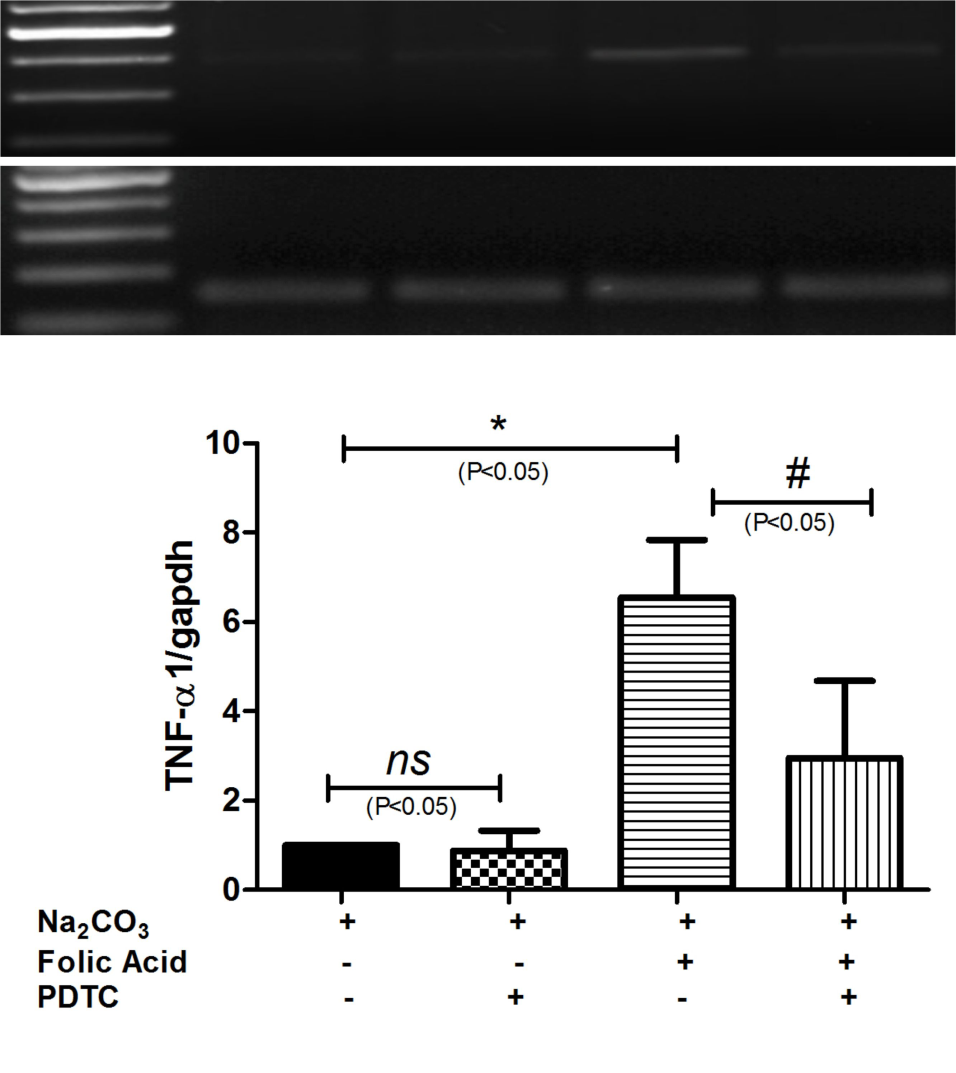
**

Supplement: S3 Fig — Effects of PDTC on TNF-α gene expression after FA induced injury in mice. The mRNA expression of TNF-α was measured at 12 hours after injection of FA+ vehicle or PDTC+FA by RT-PCR. The mRNA levels were normalized by the expression of GAPDH and were expressed as % fold change relative to control animals. Data are presented as mean ±SEM (N = 3 animals per group). *P<0.05 compared between control and FA treated group. #P<0.05 compared between FA treated group and FA+PDTC treated group. (DOCX) [file pone.0115947.s003.docx]

**Figure. S-5. Effect of PDTC on glutathione levels and redox ratio after FA induced**

**injury in mice.**


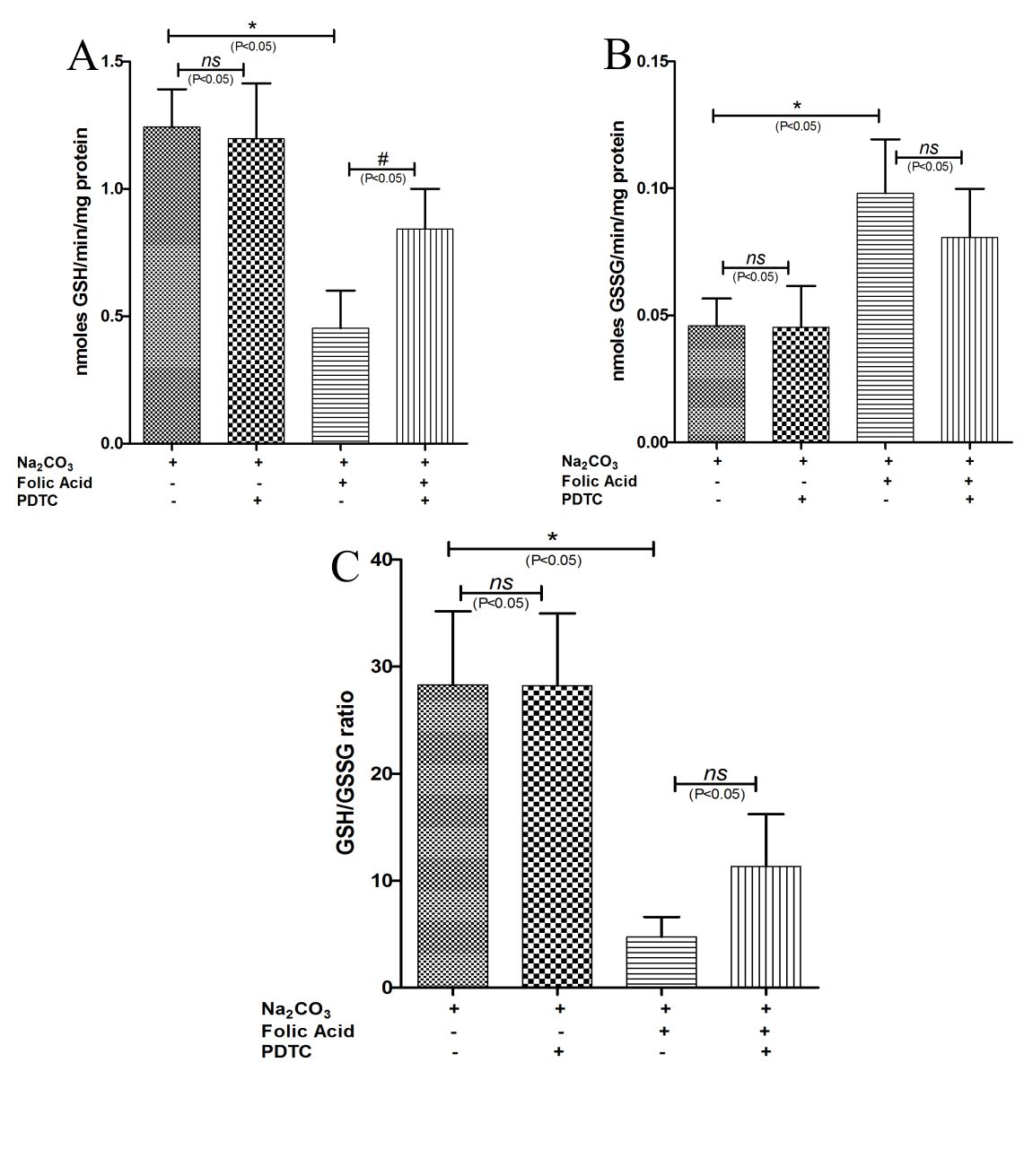

Supplement: S5 Fig — Effect of PDTC on glutathione levels and redox ratio after FA induced injury in mice. (A) GSH Levels, (B) GSSG levels and (C) redox ratio in mouse kidney homogenate of FA treated animals. Data are presented as mean ±SD (N = 6 animals per group). *P<0.05 compared between control and folic acid treated groups. #P<0.05 compared between FA treated group and FA+PDTC treated group. (DOCX) [file pone.0115947.s005.docx]

**Figure.S-6. Effects of PDTC on Pro-apoptotic gene, *Bax* expression after FA induced**

**injury in mice.**

**
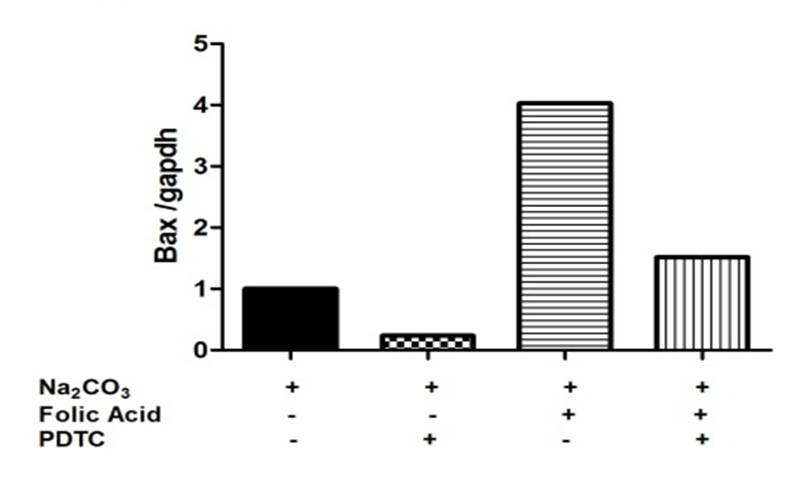
**

Supplement: S6 Fig — Effects of PDTC on Pro-apoptotic gene, Bax expression after FA induced injury in mice. The mRNA expression of Bax, was measured at 12 hrs after injection of FA+ vehicle or PDTC+FA by RT-PCR. The mRNA levels were normalized by the expression of GAPDH and were expressed as % fold change relative to control animals. Data are presented as mean ±SEM (N = 3 animals per group). *P<0.05 compared between control and FA treated group. #P<0.05 compared between FA treated group and FA+PDTC treated group. (DOCX) [file pone.0115947.s006.docx]

**Figure. S-7. Tubular injury score in FA induced injury in mice.**

**
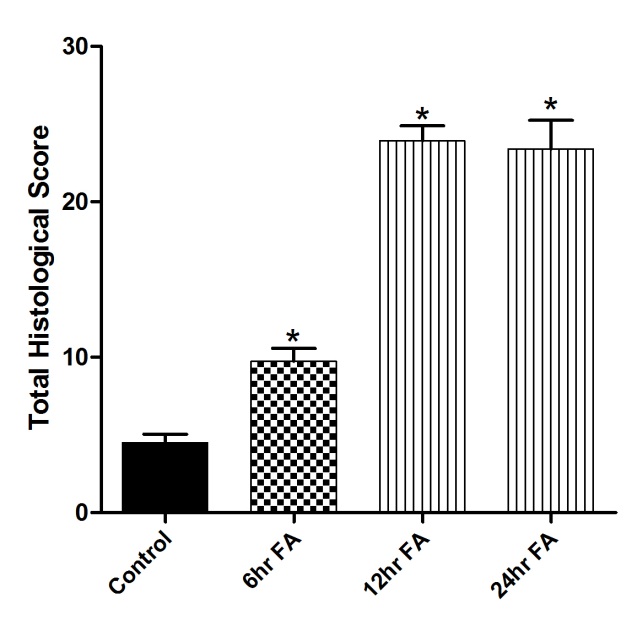
**

Supplement: S7 Fig — Tubular injury score in FA induced injury in mice. Total histological score calculated in Control and FA induced mouse kidney sections at different time point (6, 12 and 24 hrs). Semi-quantitative assessment of renal injury was presented as number of damaged tubule per total cross-sectional area of renal tissue from respective group of animals. Magnification: 200X, *P<0.05 compared between control and FA treated groups. (n = 3). (DOCX) [file pone.0115947.s007.docx]
