# Supplementary material for: The Restrained Expression of NF-kB in Renal Tissue Ameliorates Folic Acid Induced Acute Kidney Injury in Mice
Source: PLoS One. 2015 Jan 5;10(1):e115947. doi: 10.1371/journal.pone.0115947 (PMC4283964; doi:10.1371/journal.pone.0115947)

**Figure. S-4. Effect of PDTC on activity of antioxidant enzymes and ROS and LPO after FA**

**induced injury in mice.**


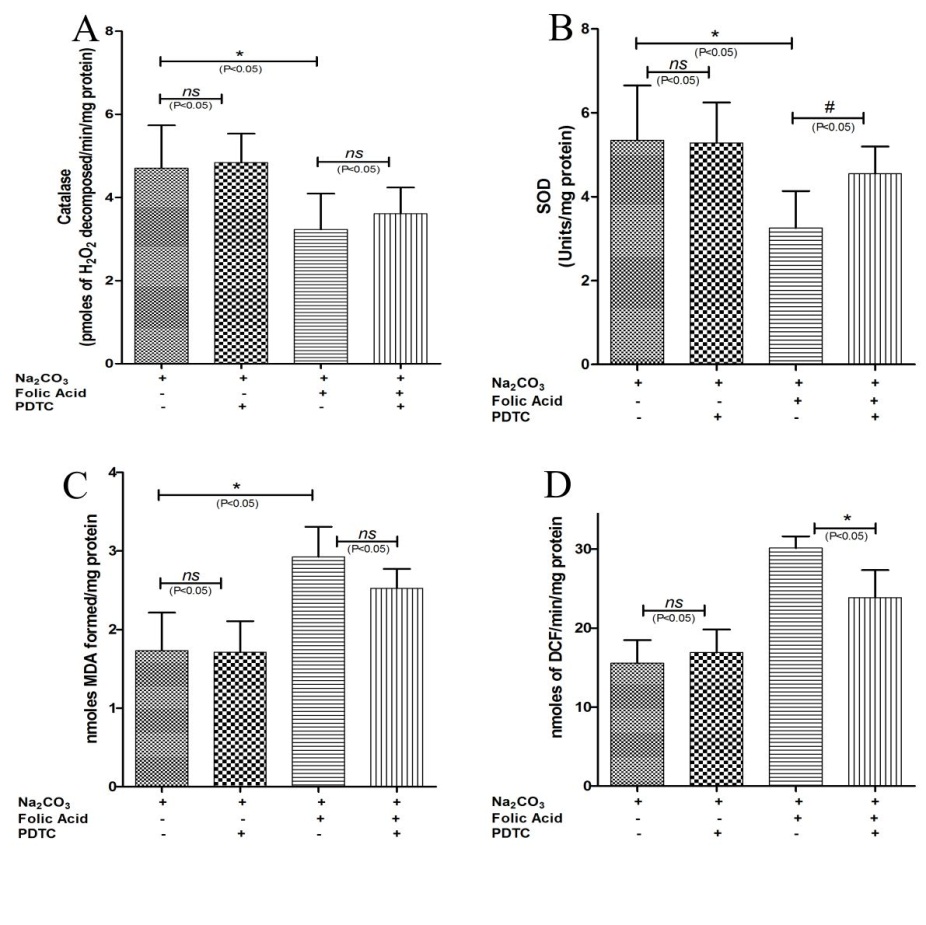

Supplement: S4 Fig — Effect of PDTC on activity of antioxidant enzymes and ROS and LPO after FA induced injury in mice. (A) SOD, (B) catalase, (C) ROS and (D) LPO in mouse kidney homogenate of FA treated animals. Data are presented as mean ±SD (N = 6 animals per group). *P<0.05 compared between control and folic acid treated groups. #P<0.05 compared between FA treated group and FA+PDTC treated group. (DOCX) [file pone.0115947.s004.docx]
